# A Widely Applicable Silver Sol for TLC Detection with Rich and Stable SERS Features

**DOI:** 10.1186/s11671-016-1442-5

**Published:** 2016-04-23

**Authors:** Qingxia Zhu, Hao Li, Feng Lu, Yifeng Chai, Yongfang Yuan

**Affiliations:** Department of Pharmacy, Shanghai 9th People’s Hospital, Shanghai Jiao Tong University School of Medicine, 280 Mo He Rd, Shanghai, 201999 China; Department of Pharmaceutical Analysis, School of Pharmacy, Second Military Medical University, Shanghai, 200433 China

**Keywords:** Non-aqueous silver sols, *N*,*N*-Dimethylformamide, Surface-enhanced Raman spectroscopy, Thin-layer chromatography

## Abstract

Thin-layer chromatography (TLC) coupled with surface-enhanced Raman spectroscopy (SERS) has gained tremendous popularity in the study of various complex systems. However, the detection of hydrophobic analytes is difficult, and the specificity still needs to be improved. In this study, a SERS-active non-aqueous silver sol which could activate the analytes to produce rich and stable spectral features was rapidly synthesized. Then, the optimized silver nanoparticles (AgNPs)-DMF sol was employed for TLC-SERS detection of hydrophobic (and also hydrophilic) analytes. SERS performance of this sol was superior to that of traditional Lee–Meisel AgNPs due to its high specificity, acceptable stability, and wide applicability. The non-aqueous AgNPs would be suitable for the TLC-SERS method, which shows great promise for applications in food safety assurance, environmental monitoring, medical diagnoses, and many other fields.

**Electronic supplementary material:**

The online version of this article (doi:10.1186/s11671-016-1442-5) contains supplementary material, which is available to authorized users.

## Background

Recently, with the popularization of portable Raman spectrometers, thin-layer chromatography (TLC) coupled with surface-enhanced Raman spectroscopy (SERS) has frequently been applied to analyze various complex systems. Analytes are isolated and preliminarily purified through the classic TLC separation method, and then, the SERS technology is used for the specific detection of trace substances on the TLC plate. Compared to other commonly used techniques, this coupling method has unique advantages such as low cost, less sample pretreatment, and high throughput of TLC separation, along with high specificity and sensitivity of SERS detection, which is suitable for preliminary screening and rapid on-site detection. Since first reported by Hezel [1], TLC-SERS has been employed for the separation and detection of various complex substances, including substituted aromatic pollutants in water samples [2], dyestuffs on works of art [3, 4], biomarkers in biological urine [5], pesticide residues from crops [6], and adulterants in botanical dietary supplements (BDS) [7, 8]. Furthermore, it has been applied in chemical synthesis analysis [9, 10] and clinical therapeutic drug monitoring (TDM) [11]. The growing number of studies has demonstrated the validity of coupling TLC with SERS and also implied its good prospect in many research fields such as food, drug, and environment.

However, it could be found that nearly all of the reported analytes in the aforementioned applications are hydrophilic and that relatively universal SERS-active sols (which are widely applicable for both hydrophilic and hydrophobic analytes) are urgently needed for the TLC-SERS method. While the aqueous sols show good enhancement for hydrophilic substances, it is difficult to detect hydrophobic analytes. Oriňák et al. [12] applied TLC-SERS technique to the analysis of hydrophobic diterpenoic acids, but the SERS spectra of the three biologically active diterpenes (gibberellic acid, abietic acid, and kaurenoic acid) showed weak and unsatisfactory Raman signals. According to the theory of dynamic surface-enhanced Raman spectroscopy (DSERS) [13–16], good SERS signals appear during the SERS substrate transformation from a wet state to a dry state. Hydrophobic analytes can hardly be trapped in aqueous nanoparticles for their incompatibility with water. Thus, enhancing SERS signals of hydrophobic analytes with aqueous sols is very difficult. To make matters worse, these SERS signals are unstable and the enhancement keeps for a short time (usually less than 60 s) due to the fast vaporization of water. Besides, SERS feature obtained by aqueous silver sols is not rich which might be overcome by inducing aggregating agents to generate high-enhancing “hot spots” [2, 4, 5, 10], but the optimization is inconvenient and sometimes inapplicable. In a word, aqueous silver sols in the aforementioned TLC-SERS applications cannot provide rich and stable SERS features.

Herein, a new SERS-active silver sol for TLC detection is reported to provide technical support for the world’s first TLC-SERS coupling instrument funded by the Chinese government and still under research for on-site rapid prescreening of adulterated drugs. After the reaction time optimization and morphology characterization, the optimal silver nanoparticles (AgNPs)-DMF sol was obtained rapidly. The newly developed AgNPs were successfully applied to test several analytes including hydrophilic and hydrophobic substances with richer spectral features (more abundant peaks) and more stable intensity than aqueous AgNP sol, which was beneficial for peak assignment and substance’s discrimination. The non-aqueous AgNPs reported herein would be suitable for the TLC-SERS method, which shows great promise to the analysis of complex systems in food safety assurance, environmental monitoring, medical diagnoses, and many other fields.

## Methods

### Materials and Apparatus

Silver nitrate; chloroauric acid; sodium citrate; polyvinylpyrrolidone (PVPK30); and all organic solvents including *N*,*N*-dimethylformamide (DMF), dimethyl sulfoxide (DMSO), methanol, ethanol, acetonitrile, and acetone of analytical grade were purchased from Sinopharm Chemical Reagent Co., Ltd., China. All reference chemicals including rosiglitazone maleate (ROS), pioglitazone hydrochloride (PIO), gliclazide (GLC), glyburide (GLB), and glipizide (GLP) were bought from National Institute for Food and Drug Control, China. While rhodamine 6G (R6G), gibberellic acid (GA), methyl orange (MO), and Sudan III (S III) were purchased from Sinopharm Chemical Reagent Co., Ltd., China. Distilled water was obtained using a Smart-DUV (18 MΩ cm resistivity) filter (Shanghai Hitech Instruments Co., Ltd., China). TLC plates (Yantai E.S.T. Silicone Tech Co., Ltd., China) consist of high-performance silica gel 60-F254 plates (silica gel particle size 8 ± 2 μm ≥80 %, layer thickness 0.2 ± 0.03 mm) with glass back plates.The plate containing a fluorescing additive, F254, was used for easy spot visualization.

Separated spots were located using an ultraviolet analyzer with 254 nm wavelength (WFH-203B, Shanghai Jing Branch Industrial Co., Ltd., China). Ultraviolet–visible (UV–Vis) absorption spectra of silver colloids were obtained with a double beam UV–Vis spectrophotometer (TU 1901, Beijing Purkinje General Instrument Co., Ltd., China). Scanning electron microscope (SEM) images were taken on a ZEISS EVO MA-10 (Carl-Zeiss, Germany). Raman spectra were recorded by a portable Raman spectrometer (BWS415, B&W Tek Inc., USA) at 785 nm, a resolution of 5 cm^−1^ and a ×20 long working distance microscope objective.

### Nanoparticle Preparation

AgNPs of water solution (AgNPs-H_2_O) were synthesized by the Lee–Meisel method [17]. Briefly, 45 mg of AgNO_3_ was dissolved in 250 mL of distilled water and heated to boiling. Five milliliters of a 1 % (*w/v*) sodium citrate tribasic solution was added to the solution under vigorous magnetic stirring and kept boiling for 1 h. AgNPs of DMF solvent (AgNPs-DMF) were prepared as followed; a mixed solution of silver nitrate (17 mg) and PVP (1:1) was added to 100 mL boiling DMF and heated for 1 min, then the AgNPs were obtained. All synthesized nanoparticles (NPs) were kept at room temperature (RT) protected from light. To obtain NPs dispersed in non-aqueous solutions, the original NPs in water were centrifuged at the speed of 9000 rpm for 10 min. The supernatants were discarded carefully, and the precipitate at the bottom was resuspended in different organic solvents.

### Sample Preparation

Analyte stock solutions were prepared by dissolving reference in optimal solvent. ROS and PIO samples were dissolved in methanol; GLB, GLP, and GLC samples were prepared in mixed solvent of methanol–chloroform (1/1, *v/v*); and the ultimate concentrations were 1 mg/mL. GA, MO, and S III stock solutions were dissolved in ethanol at a concentration of 0.1 mg/mL. Then, each analyte stock solution was ready for detection by the TLC-SERS method.

### TLC-SERS Analysis

Analyte stock solutions (1 μL) were applied to a silica gel TLC plate, let dry, and then eluted with CH_2_Cl_2_:CH_3_OH 8:1 (*v/v*). After the eluent on the TLC plate evaporated naturally, the separated spots were visualized and marked under an ultraviolet illumination at 254 nm. SERS analyses were directly performed on the plate after local deposition of 4 μL NP solution directly on the marked spot. The SERS spectra for the spots were acquired using a Raman spectrometer with a suitable power (200 mW at colorless samples and 90 mW at pigments) and an integration time of 5 s. A continuously recording mode without interval was also applied to investigate the variation discipline of the silver sol. All the measurements were repeated in triplicate. Data were pretreated with the Savitzky–Golay polynomial fitting (9-point smoothing) and baseline correction, with Matlab 7.0 (MathWorks, Massachusetts, USA) and Origin 8.0 software.

## Results and Discussion

### AgNPs-DMF Preparation and Optimization

**Fig. 1:**
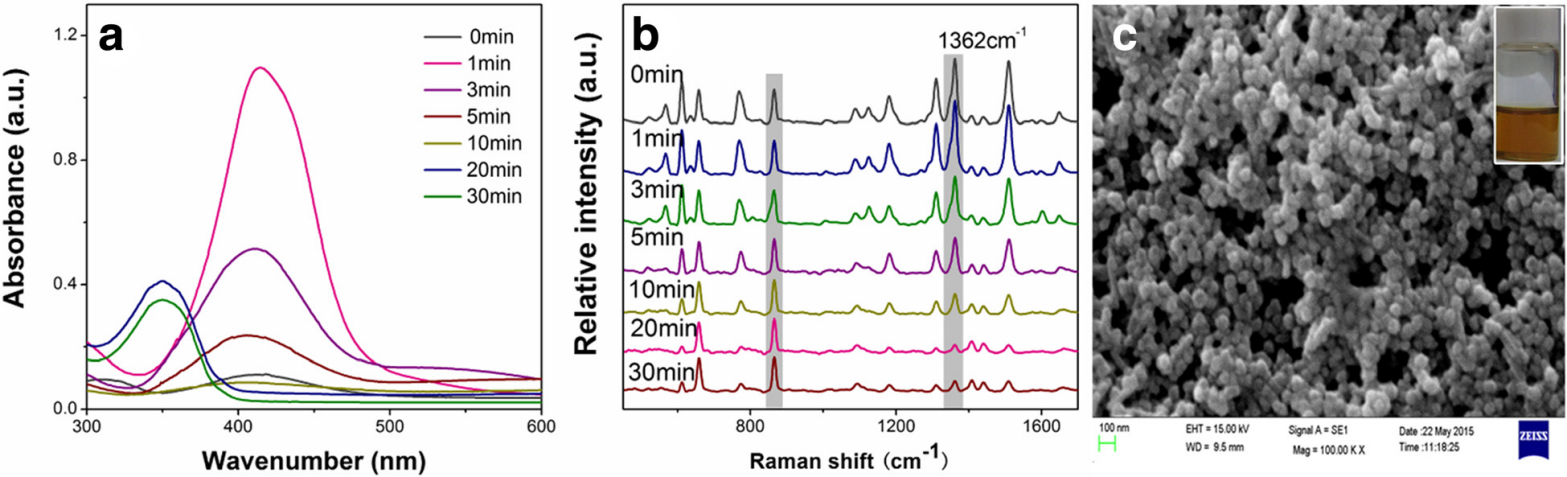
UV–Vis spectroscopy and SEM image characterization of **a** UV–Vis spectra of AgNPs-DMF obtained at different heating times and **b** corresponding R6G SERS spectra. All spectra were normalized to the 865-cm^−1^ peak. **c** SEM image of AgNPs-DMF obtained at a 1-min heating time

DMF had been used to prepare NPs in the past. However, previous reports mainly focused on synthesizing metal NPs [18–20], silver films [21], and reduced graphene oxide nanosheets [22], or studying the function of DMF [23]. The prepared SERS-active substrates had not been used for sample detection, not to mention TLC plate detection, and their SERS performance had not been developed. Here, with the experience of previous research [18–23], we provide a method of preparing a AgNPs-DMF sol that is suitable for TLC-SERS detection. The simple procedure for anisotropic AgNPs was based on the use of DMF as a solvent and also a reducing agent, in the presence of the PVP as a stabilizer. In order to evaluate the reaction conditions, a widely used SERS probe, R6G, was analyzed and the optimal conditions were selected by comparing the SERS spectra of R6G. According to Liz-Marzán’s study [18, 19, 23], a method involving Ag^+^/PVP (1:1) under reflux was preferred and the reaction times after additions of Ag^+^/PVP solutions into DMF have a great impact on NP synthesis. In this study, we investigated the SERS activities of several AgNPs after giving different heating times (e.g., 0, 1, 3, 5, 10, 20, and 30 min). The UV–Vis spectra (Fig. 1a) showed that the maximum absorption band shifted from 423 to 349 nm as the reaction time increased and the maximum absorbance increased dramatically after a heating time of 1 min and then decreased. Figure 1b shows the SERS performances of different NPs with respect to R6G. The SERS spectra obtained after a heating time of 1 min displayed the highest relative intensity at 1362 cm^−1^. SEM image of the 1-min group revealed monodisperse colloids with the diameter of AgNPs in the range of approximately 50 nm (Fig. 1c). To prove the chemical stability of the fastly prepared AgNPs, SERS sensitivity of the 1-min-prepared AgNPs within 2 months was investigated (Additional file 1: Figure S1a). SERS intensity of R6G remained at the same level in 60 days (Additional file 1: Figure S1b), which indicated the good stability of the PVP-protected AgNPs. TEM image and UV–Vis spectra characterization results (Additional file 1: Figure S2) were also provided to interpret the temporal stability of the SERS substrate. Most of the freshly prepared AgNPs were spherical while part of them transformed into nanoprism, which was good for generating a “hot spot” due to the anisotropic shape. The particles distributed uniformly (Additional file 1: Figure S2a), and many groups of NPs scattered in the PVP background (Additional file 1: Figure S2b). Protected by the PVP, the movement of AgNPs had been restricted, resulting in a limited agglomeration. Thus, after being stored for 60 days, only part of the particles gathered slightly (insert of Additional file 1: Figure S2b). As depicted in the UV–Vis spectra (Additional file 1: Figure S2c), there was no significant change in the maximum absorption band with a slightly broaden peak, which indicated a similar particle size and relatively uneven distribution after being stored for 2 months. Additional file 1: Figure S2 further proves the stability of the nanostructure within 60 days, which ultimately led to the temporal stability of SERS detection (in our opinion and according to the actual requirement, 60 days is basically enough for a common TLC-SERS experiment). Thus, 1 min was selected as the optimum heating time for preparing AgNPs-DMF in subsequent experiments. The preparation process was much faster compared to the traditional Lee–Meisel method which usually lasted more than 1 h.

### Richer Spectral Features Provided by the AgNPs-DMF Sols

**Fig. 2:**
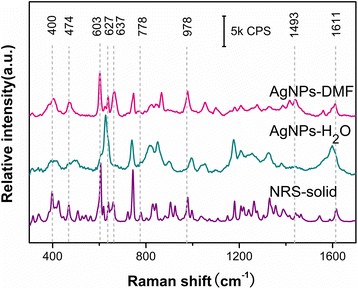
TLC-SERS spectra of ROS by AgNPs-DMF and AgNPs-H_2_O and the normal Raman spectra (NRS) of the solid

The optimized AgNPs-DMF sols were applied to detect several commonly adulterated hypoglycemic drugs which had been reported by aqueous AgNPs in the previous study [24]. We found that the SERS spectra got by the AgNPs-DMF groups held richer spectral features than those of the AgNPs-H_2_O groups (ROS, for example). Figure 2 presents the comparison result of the spectra by the two groups. By collecting at the optimum detection time, the AgNPs-DMF group showed more abundant peaks (400, 474, 603, 627, 637, 778, 978, 1493, 1611 cm^−1^) which may be due to its better affinity with AgNPs in DMF than in H_2_O [24]. Richer spectral features would be beneficial for the discrimination of substance especially for the resolution of quite similar SERS spectra from structural analogues [24]. It also can be found that the hit quality index (HQI) value of the SERS spectra by AgNPs-DMF (0.74) with the normal Raman spectrum (NRS) in the fingerprint region 550~850 cm^−1^ was higher than that of AgNPs-H_2_O (0.37). The AgNPs-DMF group presented higher similarity to the NRS which was beneficial for peak assignment of ROS. Similar results can be got for the other three analytes. As we know, in conventional SERS measurement, the SERS spectral feature was much different from the NRS, in both the relative intensity and the peak position. Here, the AgNPs-DMF group presented higher similarity to NRS than that of the AgNPs-H_2_O group, which might be contributed by the following two reasons [25, 26]. Firstly, most analytes showed better affinity with AgNPs in DMF than in H_2_O; thus, the chemical interaction (between the analyte’s molecule and Ag surface) which might lead to a change of the symmetry or even the electronic structure of the molecule was weaker and ultimately resulted in less change of the relative intensity and frequency shift. Secondly, due to the surface selection rule determined by the electromagnetic field on the Ag surface, it will selectively enhance the vibrational mode close to the surface. When AgNPs-DMF was applied, better affinity resulted in similar vibrational mode; thus, the SERS spectra of the AgNPs-DMF group would be much similar to that of the NRS. Richer features and higher similarity to NRS guaranteed the better specificity of AgNPs-DMF in the TLC-SERS method though DMF signals’ interferences existed.

**Fig. 3:**
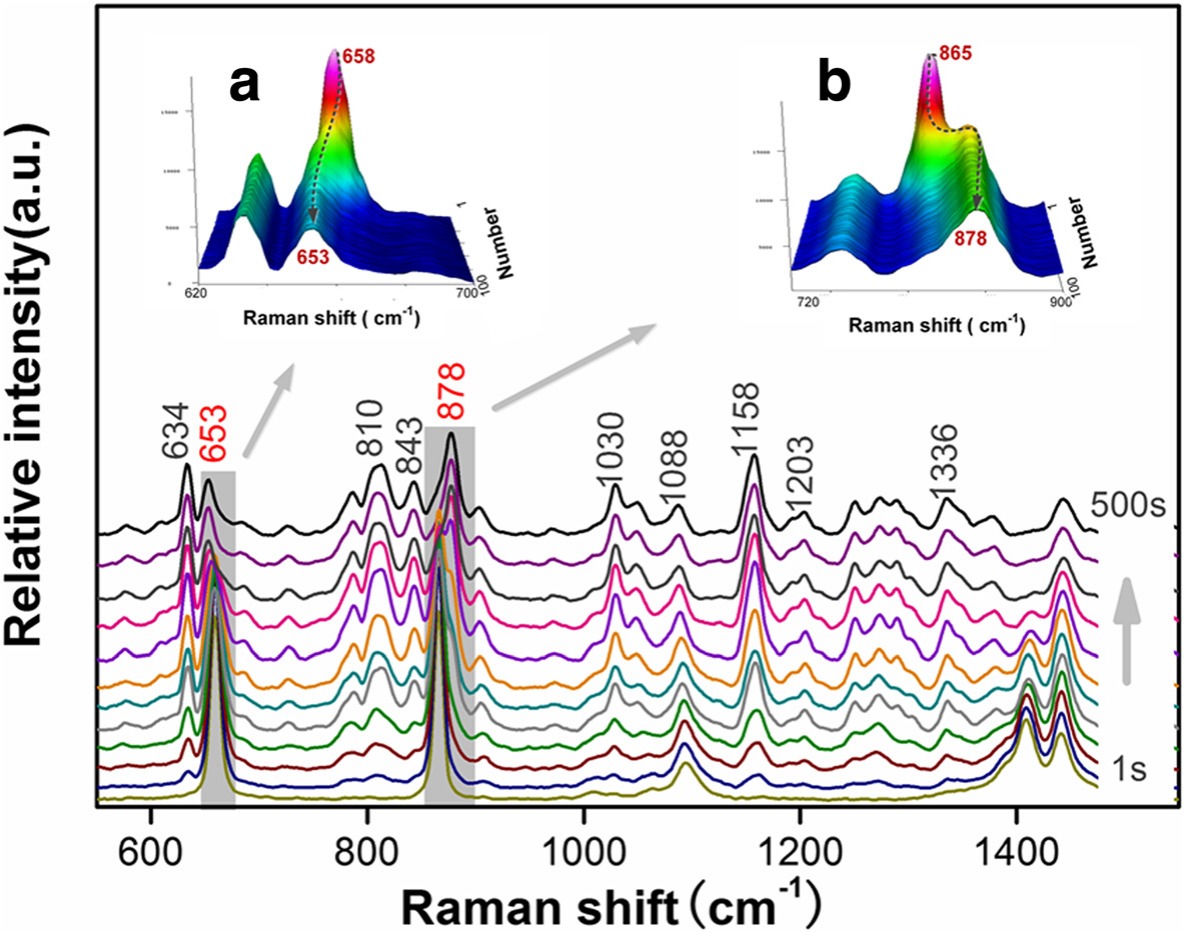
Series of time-dependent SERS spectra of GLP. The *inserts* represent the magnified spectral ranges of **a** 620–700 and **b** 720–900 cm^−1^

As we know, most solvents held strong Raman features except for water. In this study, when the AgNPs-DMF sols were used, Raman signals (320, 353, 404, 659, 865, 1093, 1049, 1440, and 1663 cm^−1^) from DMF solvent (Additional file 1: Figure S3) existed especially at the beginning of the detection. But the interference from DMF signals can be partially eliminated by the time-dependent detection, for instance with hydrophobic substance GLP (Fig. 3). By recording a series of SERS spectra after dropping AgNP sol onto the TLC spot, we found that signals from the GLP gradually appeared (634, 653, 787, 810, 843, 878, 1030, 1088, 1158, 1203, 1336, 1442, 1530, 1582 cm^−1^) and the intensity increased slowly as time elapsed, while the intensity of DMF signals gradually decreased. The variation ultimately reached a plateau when most SERS signals presented in the spectra were from the analyte rather than DMF solvent; then, DMF interference can be ignored to a certain degree. For example, DMF showed a stable signal at 865 cm^−1^, which was very close to the 878-cm^−1^ peak of GLP. As the detection went on, a time-resolved process could be observed clearly (insert b of Fig. 3); an 878-cm^−1^ peak increased while an 865-cm^−1^ peak declined; the two peaks were differentiated ultimately during the variation. A similar phenomenon could also be observed at 658 and 653 cm^−1^, which differed by only 5 cm^−1^ (insert a of Fig. 3).

More abundant signals obtained by AgNPs-DMF provided more features for peak assignment and discrimination, which also made up for the interference from DMF signals that might cover some SERS peaks of the analyte, and ultimately resulted in higher specificity compared to commonly used silver sols. The ability of distinguishing two close peaks further illustrated its high specificity, and the presence of DMF signals would have little influence with time-dependent detection mode, which will be in the following section.

### More Stable Intensity Provided by the AgNPs-DMF Sols

**Fig. 4:**
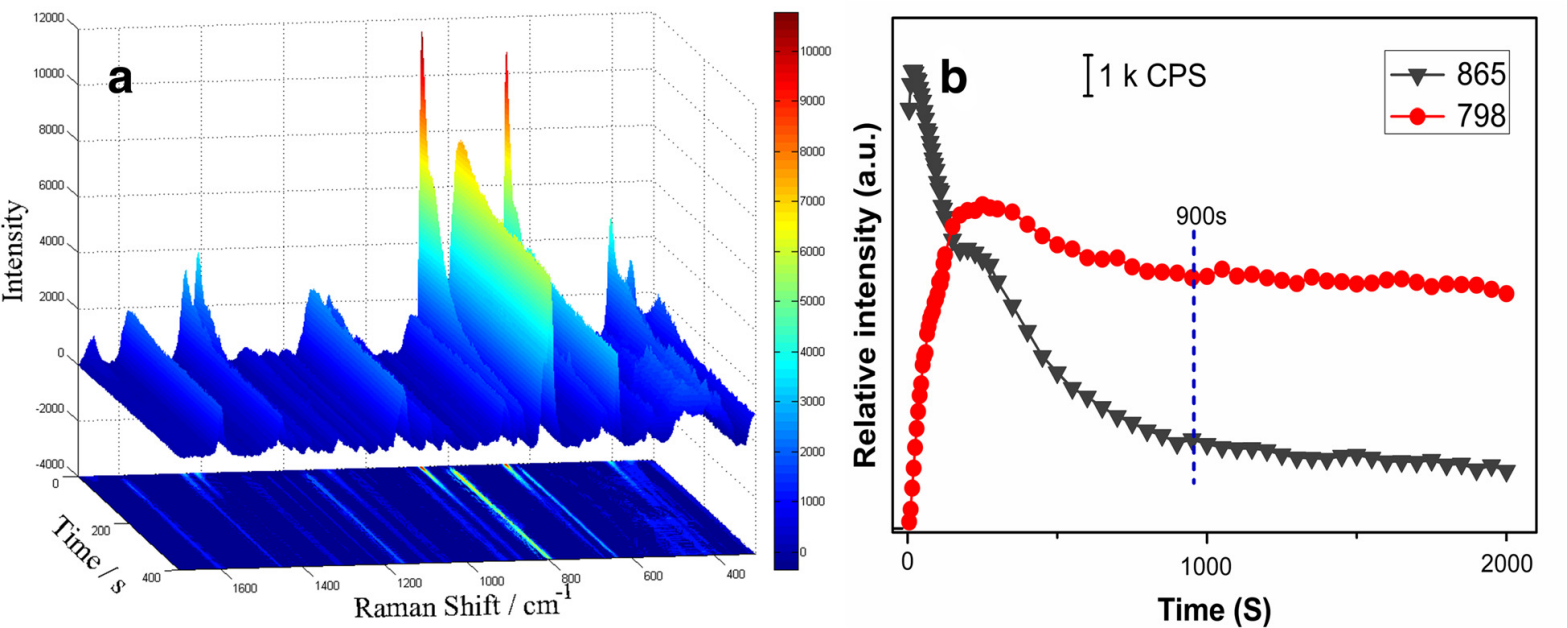
Long stable period provided by the AgNPs-DMF sols. **a** TLC-SERS spectra of GLC on AgNPs-DMF as a function of laser irradiation time. A corresponding contour plot is shown under the SERS spectra. **b** Relationship between exposure time and relative intensity of the 789-cm^−1^ band and 865-cm^−1^ band

According to the theory of DSERS [13–15], good SERS signals appeared when the SERS substrate transformed from a wet state to a dry state. A relatively long stable period of SERS enhancement would be propitious for good signal collection. To further study the SERS performance of AgNPs-DMF, the SERS spectra obtained by continuously recording were analyzed to investigate this crucial factor in SERS detection. Figure 4a shows a series of SERS spectra of GLC on AgNPs-DMF as a function of laser irradiation time. The detection was conducted continuously for 33 min to better evaluate the stability. Interestingly, bands from GLC gradually appeared with a slow increase in intensity as the laser irradiation time increased, while the intensity of DMF bands decreased slowly, but the variation rates for bands from GLC and DMF were different. Obviously, as depicted in the contour plot of Fig. 4a, the increases in the intensities of the bands at 633, 798, and 813 cm^−1^ are faster than the reduction in the intensities of the bands at 658 and 865 cm^−1^, and the variation ultimately reached a plateau at the 900th second (15th minute). In the stable period, the SERS spectra of GLC showed abundant strong features of objective substance with weak DMF signals, which would be beneficial for discrimination. GLC was also tested with silver sols condensed by factors of one, five, and ten (Additional file 1: Figure S4). The relationship between time and the relative intensity of peaks at 865 cm^−1^ from DMF and 798 cm^−1^ from GLC presented that all the three groups tended to be stable at around the 15th minute, which indicated that the abovementioned variation discipline would not be influenced by AgNP concentration.

**Fig. 5:**
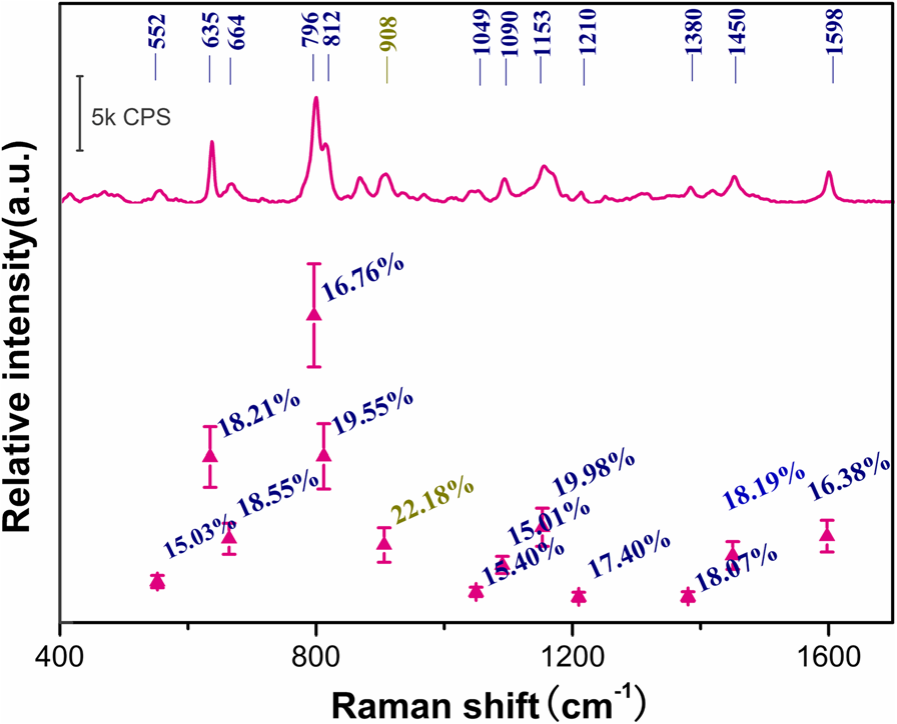
Error bars and RSD result of intensities of main peaks from GLC

To better display the abovementioned time-dependent variation discipline of AgNPs-DMF in the TLC-SERS detection, the relationship between time and relative intensity (peaks at 865 and 798 cm^−1^ for example) is displayed in Fig. 4b. The intensity of the DMF signal at 865 cm^−1^ from DMF decreased slowly as that of the 798-cm^−1^ peak from the analyte increased gradually and ultimately reached the stable period. Amazingly, the enhancement lasted more than half an hour (compared to less than 60 s from common silver sol) with no significant decrease in the intensity of GLC peaks, while signals from DMF were relatively weak and still decreased slowly. However, when AgNPs were dispersed in other common solvents (H_2_O, DMSO, acetonitrile, methanol, ethanol, and acetone), SERS signals varied differently without a relatively long stable period. The seven groups of AgNPs were employed in the TLC-SERS detection of the GLC sample spots; the relationship between time and relative intensity of peaks 798 cm^−1^ was displayed in Additional file 1: Figure S5. It seemed that the acetone and water groups held poor SERS enhancement to GLC. The methanol, ethanol, and acetonitrile groups presented a rapid rise and then slowly decline tendency, while the DMF and DMSO groups increased gradually and ultimately reached a plateau. For the DMSO group, the strong solvent bands interfered with the identification of GLC. The variation tendency of the DMF group obtained by pretreatment was similar to that of the AgNPs-DMF synthesis while the time needed to reach a plateau was different, which might be related to the sophisticated pretreatment. Though the phenomenon of Additional file 1: Figure S5 may be a complex influence of polarity, volatility, refractive index, viscosity, etc., and needs to be further explored, the comparison result further demonstrated that DMF was the preferable solvent for dispersing AgNPs due to its relatively long stable period which guaranteed the optimum collection time. The time-dependent collection was repeated in triplicate, by selecting spectra at the 900th second during the platform period; the relative standard deviations (RSDs) of the relative intensities of the main peaks present in the SERS spectrum of GLC are given in Fig. 5. Apart from individual peaks at 908 cm^−1^, the RSD values of the peaks were all lower than 20 %, which indicated acceptable reproducibility as far as TLC-SERS was concerned. The AgNPs-DMF sol reported herein showed better stability, which would improve the robustness of the following TLC-SERS method.

### Wider Applicability of the AgNPs-DMF Sols

In the previous TLC-SERS studies [2–8], aqueous silver sols prepared through the classical Lee–Meisel method were the most widely used sols, and most applications are hydrophilic substances. Actually, a lot of analytes in great testing demand were hydrophobic, but this type of aqueous AgNP sols showed poor SERS performance to them, for example, the antidiabetic drug gliclazide (GLC) (Additional file 1: Figure S6). Poor SERS signals of GLC can be obtained by the original aqueous silver sols (Additional file 1: Figure S6a); an aggregating agent (KNO_3_) which can induce nanoparticle aggregation to improve ameliorating the result [27] was also employed, but little improvement (a relative intensity of 3030 at 798 cm^−1^) of SERS signals was observed even though the proportion of AgNPs and KNO_3_ had been optimized (Additional file 1: Figure S6b), which indicated that aqueous AgNP sols held poor TLC-SERS enhancement to hydrophobic analytes. To expand the application of the TLC-SERS method, SERS-active sols with high universality to not only hydrophilic but also hydrophobic analytes were needed.

**Fig. 6:**
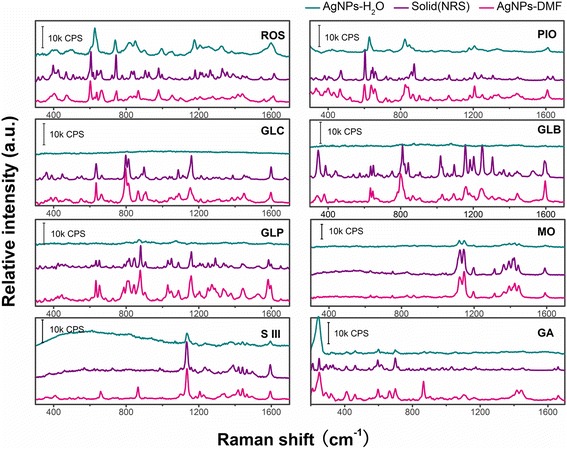
TLC-SERS spectra of different analytes and normal Raman spectra (NRS) of their solid

To evaluate the universality of AgNPs-DMF in the TLC-SERS method, the sols were applied to the detections of both the hydrophilic and hydrophobic analytes from various fields including drugs (ROS, PIO, GLC, GLB, and GLP), pigments (MO and S III), and hormones (GA). These analytes were water-insoluble substances except for the hydrophilic ROS and PIO of the five easily adulterated hypoglycemic drugs. All the analytes were in great demand of fast on-site detection; among them, MO and S III were toxic artificial pigments usually being illegally added in foods, while GA was pesticide residue in crops. A comparison of results obtained with the commonly used aqueous AgNP sols is shown in Fig. 6. All the eight hydrophilic and hydrophobic analytes of different fields were detected successfully, which indicated good universality of AgNPs-DMF. The hydrophilic ROS and PIO can be enhanced by both AgNPs-DMF and AgNPs-H_2_O, while SERS spectra obtained by AgNPs-DMF presented more peaks for identification. AgNPs-DMF showed better SERS performance with more SERS features, while poor SERS spectra were obtained by AgNPs-H_2_O. It showed nearly no enhancement for GLB and GLP, which was similar to our previous result for GLC (Additional file 1: Figure S6), and the two commonly used SERS probes (MO and S III) showed relatively weak signals compared to AgNPs-DMF as well. For GA, which had been reported to show poor TLC-SERS signals with aqueous AgNPs [12], more features, especially characteristic peaks (209, 248, 290, and 544 cm^−1^) at low wave numbers, were observed when AgNPs-DMF was used. The result implied the highly universal AgNPs-DMF for TLC-SERS detection was suitable for various hydrophilic and hydrophobic substances involving drugs and food safety assurance, environmental monitoring, and many other fields.

## Conclusions

In this study, a non-aqueous silver sol for TLC detection with rich and stable SERS features was successfully developed and employed in the analysis of both hydrophobic and hydrophilic analytes. The SERS-active sols prepared herein presented not only richer spectral features for peak assignment which was beneficial for structural analysis and analogue discrimination but also more stable features which would improve the robustness of the TLC-SERS method. What is more is that the applications could be expanded due to the higher universality of the non-aqueous silver sol. To provide better technical support for the TLC-SERS coupling instrument, additional work is currently ongoing in our lab to further optimize the preparation process of the AgNPs-DMF to improve the repeatability and the limits of detection and also to expand the application of the TLC-SERS method in the analysis of complex systems in drug safety, environmental monitoring, and many other fields.

## Additional file

Additional file 1: Figure S1. (a) TLC-SERS spectra of 1 × 10^−9^ M R6G measured in 60 days. (b) The normalized Raman intensities of R6G Raman peak at 1362 cm^−1^ as a function of the measurement time. **Figure S2.** (a) TEM image of the freshly prepared AgNPs (Day 0). (b) TEM image of the AgNPs stored for 60 days (Day 60), Arrows presented the aggregation of different degrees. (c) UV–Vis spectra of the freshly prepared AgNPs and the AgNPs stored for 60 days. **Figure S3.** Raman spectrum of DMF solvent. **Figure S4.** Relationship between time and relative intensity of characteristic peaks at 789 cm^−1^ and 865 cm^−1^ in SERS spectra of GLC obtained by AgNPs-DMF condensed by factors of 1, 5, and 10. **Figure S5.** Variations in intensities of the main bands at 798 cm^−1^ in SERS spectra of GLC on different AgNPs as a function of exposure time. **Figure S6.** (a) NRS of GLC and SERS spectra of GLC obtained with different silver sols. (b) SERS spectra obtained by AgNPs and KNO_3_ with various volume ratios. (DOC 823 kb)
