# Supplementary material for: A Widely Applicable Silver Sol for TLC Detection with Rich and Stable SERS Features
Source: Nanoscale Res Lett. 2016 Apr 23;11:220. doi: 10.1186/s11671-016-1442-5 (PMC4842203; doi:10.1186/s11671-016-1442-5)
Supplement: Additional file 1: Figure S1. — (a) TLC-SERS spectra of 1 × 10^−9^ M R6G measured in 60 days. (b) The normalized Raman intensities of R6G Raman peak at 1362 cm−1 as a function of the measurement time. Figure S2. (a) TEM image of the freshly prepared AgNPs (Day 0). (b) TEM image of the AgNPs stored for 60 days (Day 60), Arrows presented the aggregation of different degrees. (c) UV–Vis spectra of the freshly prepared AgNPs and the AgNPs stored for 60 days. Figure S3. Raman spectrum of DMF solvent. Figure S4. Relationship between time and relative intensity of characteristic peaks at 789 cm−1 and 865 cm−1 in SERS spectra of GLC obtained by AgNPs-DMF condensed by factors of 1, 5, and 10. Figure S5. Variations in intensities of the main bands at 798 cm−1 in SERS spectra of GLC on different AgNPs as a function of exposure time. Figure S6. (a) NRS of GLC and SERS spectra of GLC obtained with different silver sols. (b) SERS spectra obtained by AgNPs and KNO3 with various volume ratios. (DOC 823 kb) [file 11671_2016_1442_MOESM1_ESM.doc]

**Additional file 1**

**A widely applicable silver sol for TLC detection with rich and stable SERS features**

Qingxia Zhu ^1^, Hao Li ^2^, Feng Lu ^2^, Yifeng Chai ^2^, Yongfang Yuan ^1*^

^1^ Department of Pharmacy, Shanghai Ninth People's Hospital, Shanghai Jiao Tong University School of Medicine, Shanghai, 201999, China.

^2^ Department of Pharmaceutical Analysis, School of Pharmacy, Second Military Medical University, Shanghai 200433, China.

* Correspondence: nmxyyf@126.com

**
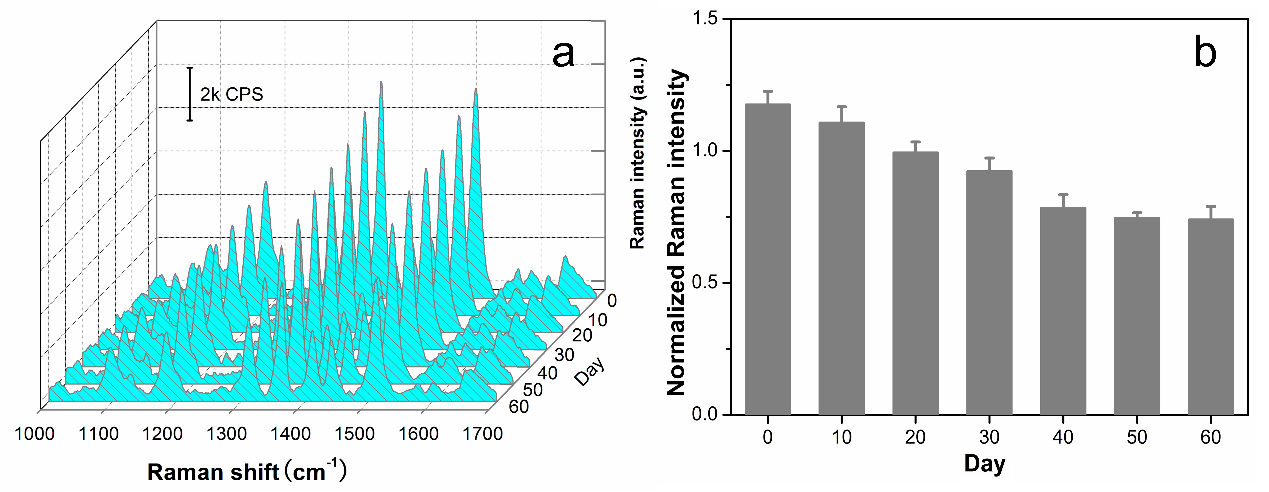
**

**Figure S1** (a) TLC-SERS spectra of 1ⅹ10^-9^ M R6G measured in 60 days; (b) The normalized Raman intensities of R6G Raman peak at 1362 cm^−1^ as a function of the storage time.


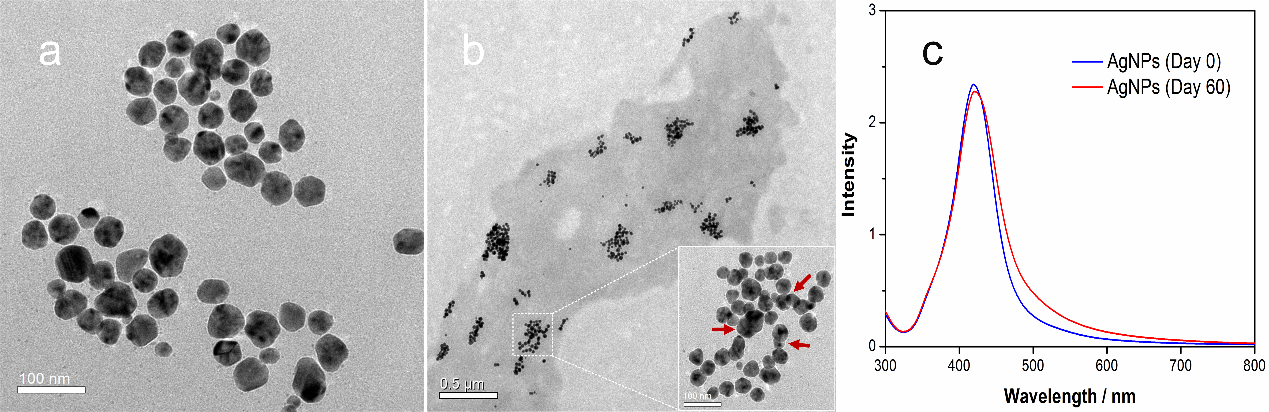


**Figure S2** (a) TEM image of the freshly prepared AgNPs (Day 0); (b) TEM image of the AgNPs stored for 60 days (Day 60), Arrows presented the aggregation of different degrees; (c) UV-vis spectra of the freshly prepared AgNPs and the AgNPs stored for 60 days.


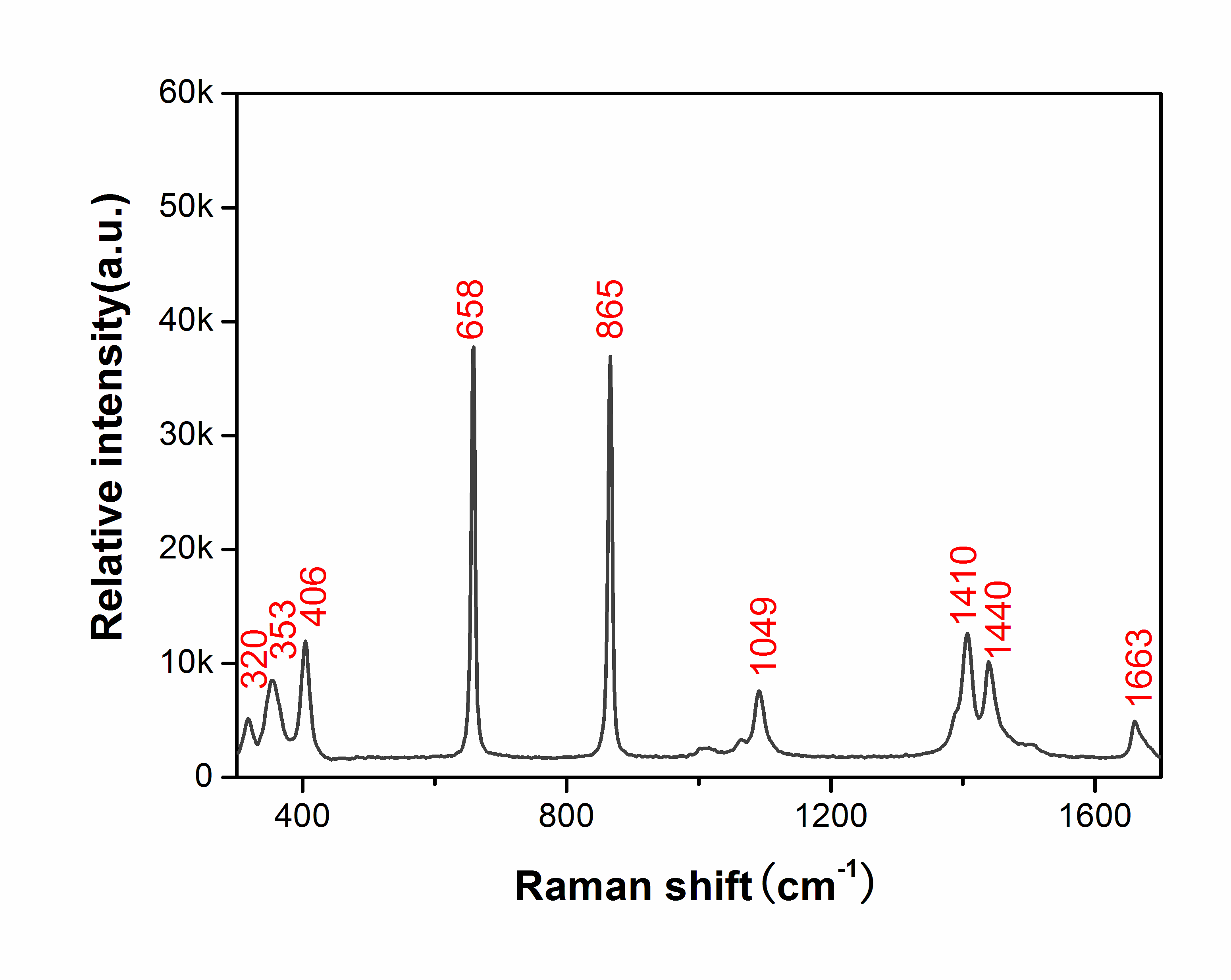


**Figure S3** Raman spectrum of DMF solvent.


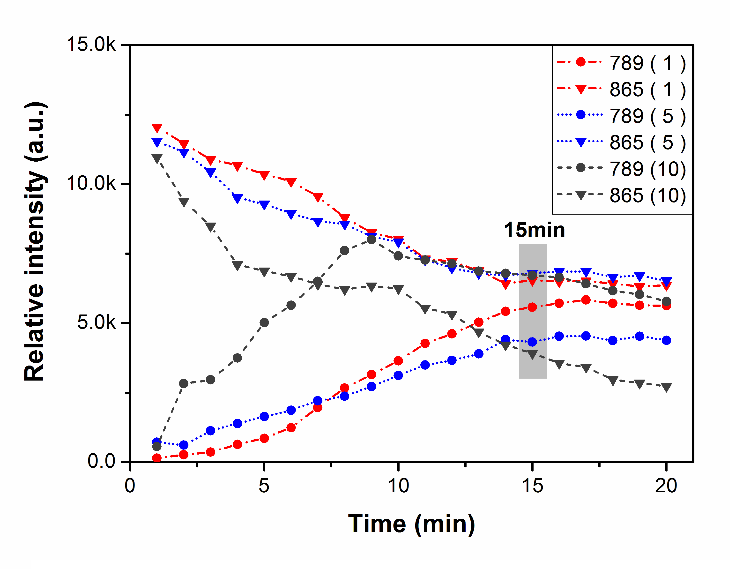


**Figure S4** Relationship between time and relative intensity of characteristic peaks at 789 cm^−1^ and 865 cm^−1^ in SERS spectra of GLC obtained by AgNPs-DMF condensed by factors of 1, 5, and 10.

**
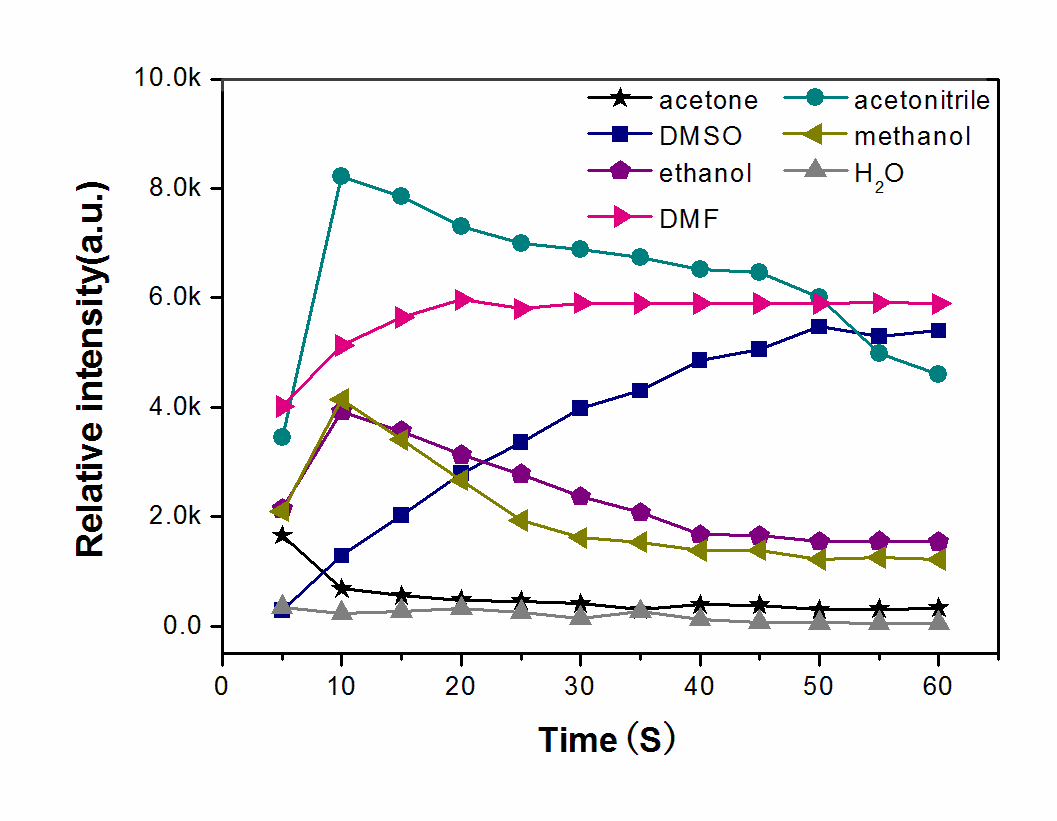
**

**Figure S5** Variations in intensities of the main bands at 798 cm^−1^ in SERS spectra of GLC on different AgNPs as a function of exposure time.


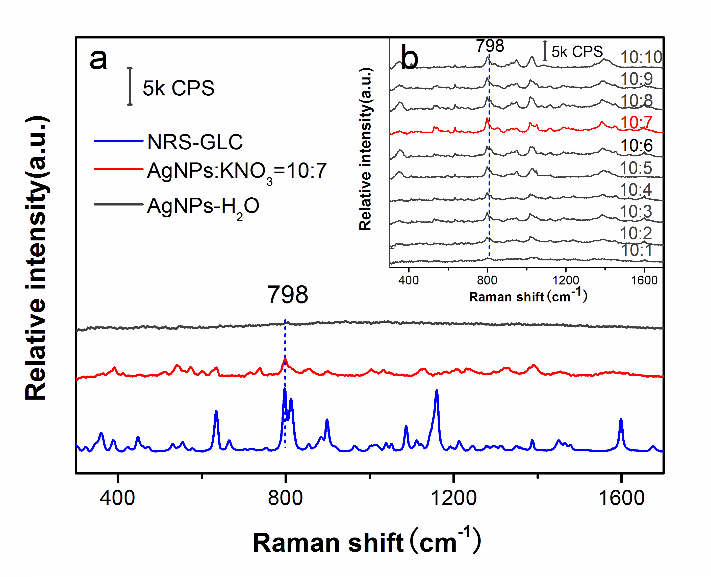


**Figure S6** (a) NRS of GLC and SERS spectra of GLC obtained with different silver sols; (b) SERS spectra obtained by AgNPs and KNO_3_ with various volume ratios.
